# Modification of EGF-Like Module 1 of Thrombospondin-1, an Animal Extracellular Protein, by O-Linked N-Acetylglucosamine

**DOI:** 10.1371/journal.pone.0032762

**Published:** 2012-03-05

**Authors:** Brian R. Hoffmann, Yuanyuan Liu, Deane F. Mosher

**Affiliations:** Departments of Medicine and Biomolecular Chemistry, University of Wisconsin, Madison, Wisconsin, United States of America; Center for Cancer Research, National Cancer Institute, United States of America

## Abstract

Thrombospondin-1 (TSP-1) is known to be subject to three unusual carbohydrate modifications: C-mannosylation, O-fucosylation, and O-glucosylation. We now describe a fourth: O-β-N-acetylglucosaminylation. Previously, O-β-N-acetylglucosamine (O-β-GlcNAc) was found on a threonine in the loop between the fifth and sixth cysteines of the 20^th^ epidermal growth factor (EGF)-like module of *Drosophila* Notch. A BLAST search based on the *Drosophila* Notch loop sequence identified a number of human EGF-like modules that contain a similar sequence, including EGF-like module 1 of TSP-1 and its homolog, TSP-2. TSP-1, which has a potentially modifiable serine in the loop, reacted in immuno-blots with the CTD110.6 anti-O-GlcNAc antibody. Antibody reactivity was diminished by treatment of TSP-1 with β-N-acetylhexosaminidase. TSP-2, which lacks a potentially modifiable serine/threonine in the loop, did not react with CTD110.6. Analysis of tandem modules of TSP-1 localized reactivity of CTD110.6 to EGF-like module 1. Top-down mass spectrometric analysis of EGF-like module 1 demonstrated the expected modifications with glucose (+162 Da) and xylose (+132 Da) separately from modification with N-acetyl hexosamine (+203 Da). Mass spectrometric sequence analysis localized the +203-Da modification to Ser580 in the sequence ^575^CPPGYSGNGIQC^586^. These results demonstrate that O-β-N-acetylglucosaminylation can occur on secreted extracellular matrix proteins as well as on cell surface proteins.

## Introduction

**Figure 1:**
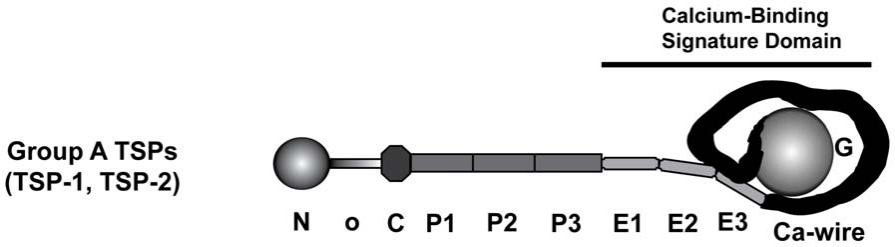
Schematic diagram of Group A TSPs modular composition. Group A TSPs, TSP-1 and -2, are trimeric, multi-modular calcium-binding proteins. The subunit comprises a N-terminal module (N), oligomerization sequence (o), von Willebrand Factor C module (C), three properdin-like modules (P123), three EGF-like modules (E123), a calcium-binding wire (Ca), and a globular lectin-like C-terminal module (G).

Thrombospondins (TSPs) are large secreted, calcium-binding glycoproteins. There are 5 TSPs in humans: 2 trimeric Group A TSPs (TSP-1 and TSP-2) and 3 pentameric Group B TSPs (TSP-3, TSP-4, TSP-5) [1]. Group A TSPs are composed of an N-terminal module (N), an oligomerization sequence (o) that is responsible for trimer formation, a von Willebrand Factor C module (C), three properdin-like modules (P123), three epidermal growth factor (EGF)-like modules (E123), a calcium-binding (Ca) wire, and a globular lectin-like C-terminal module (G) (Figure 1) [1]. Group B TSPs lack the C and P123 modules and contain an extra EGF-like module [1]. TSPs are widely distributed in connective tissues, platelets, blood vessels, and neuromuscular tissues and modulate numerous processes through interactions with extracellular matrix (ECM) components and cell surface receptors [2], [3].

TSPs are known to be subject to three unusual carbohydrate modifications. Group A TSPs are C-mannosylated on the tryptophans of WXXW sequences in the properdin-like modules, as are other proteins with similar sequences [4], [5]. The properdin-like modules also contain a CSX(S/T)C sequence that is O-fucosylated by O-fucosyltransferase 2, which then undergoes glucose addition, and is critical for protein maturation [4], [6], [7]. O-glucosylation, as in a number of EGF-like modules containing a CXSXPC sequence between the first and second cysteines, occurs in the 1^st^ EGF-like module (E1) in TSP-2 [8] and presumably in TSP-1 and TSP-4. The TSP-2 E1 module also has a N-glycosylated NGT sequence between the fifth and sixth cysteines [1]. Since extracellular EGF-like modules in *Drosophila* Notch have recently been identified to contain O-linked β-N-acetylglucosamine (O-β-GlcNAc) [9], we set out to identify any possible O-β-GlcNAc modification of TSP-1 EGF-like modules. We now report a fourth unusual modification of TSP-1: O-linked β-N-acetylglucosamine (O-β-GlcNAc).

## Materials and Methods

### Purification of platelet TSP-1

TSP-1 was purified from releasate of thrombin-activated platelets by heparin-agarose affinity chromatography followed by gel exclusion chromatography as described previously [10]. The protocol was modified to utilize fast performance liquid chromatography with HiTrap Heparin and Superose12 (GE Healthcare). Proteins were in Tris-buffered saline (TBS, 10 mM Tris, 150 mM sodium chloride, pH 7.4) containing 0.3 or 2 mM calcium chloride. Concentration of TSP-1 was determined by absorbance at 280 nm [11], [12].

### Expression of recombinant full length TSPs or modular constructs

The pAcGP67.coco (COCO) vector was used to produce recombinant baculovirus with which to infect insect High Five cells cultured in SF-900 medium (InVitrogen). The viruses directed expression of His-tagged TSP-derived constructs as secreted proteins that could be purified facilely from conditioned medium. This method has been shown to produce native, glycosylated, and functional TSP modules [8], [11], [12], [13], [14], [15]. Protein concentration was determined by absorbance as with pTSP-1. The following is the sequence of the construct that contained the E1 module of TSP-1 (underlined) and was subjected to MS mass determination and manipulation: ADPDGCLSNPCFAGVKCTSPDGSWKCGACPPGYSGNGIQCTLELVPRGSAAGHHHHHH.

### Anti-GlcNAc Immuno-blotting and SDS-PAGE

TSP protein samples were treated with SDS-PAGE loading buffer, subjected to polyacrylamide gel electrophoresis with a 3.3% stacking and 10% separating gel, and stained with gel code blue or immuno-blotted as described previously [16]. CTD110.6 mouse monoclonal antibody for O-β-GlcNAc purchased as ascites (Abcam) and peroxidase-conjugated goat anti-mouse IgG (Jackson ImmunoResearch Laboratory) were diluted, respectively, 1∶1000 and 1∶20000 in 0.05% Tween-20 in TBS. Kaleidoscope Prestained Molecular Weight Markers (BioRad) were used as size determinants. Some TSP-1 samples were incubated with 0.1 U/µL of the recombinant protein fusion of β-N-acetyl-hexosaminidase and maltose binding protein (New England BioLabs) in an equal part of 50 mM sodium citrate, pH 4.5, for 1 hr at 37°C for removal of O-linked N-acetyl hexosamine (O-HexNAc) adducts [9] before immuno-blotting.

### Linear Trap Quadrapole Fourier Transform Ion Cyclotron Resonance (LTQ-FT ICR) MS Analysis

Top-down (LTQ-FT ICR) MS analysis (UW-Madison Human Proteomics Facility) was performed to look for post-translational modifications of TSP-1 EGF-like modules. Recombinant E1 of TSP-1 was dialyzed into 0.1% acetic acid and then diluted in appropriate buffers to achieve final concentrations of 1% acetic acid and 30–40% acetonitrile. Collision-induced dissociation energy was used at q = 7. Modified and unmodified peaks were summed and percent of modification was determined as modified/total ×100.

### Matrix-Assisted Laser Desorption Ionization Time-of-Flight/Time-of-Flight (MALDI-TOF/TOF) MS

TSP-1 modular constructs were reduced in 10 mM dithiothreitol at 56°C for 1 hr, cooled to 22°C, alkylated with 20 mM iodoacetamide at 22°C for 30 min in the dark, and trypsinized at a 20∶1 substrate∶protease ratio using sequence grade modified trypsin (Promega, Cat. #V511A) for 16–20 hours. The Bruker ULTRAFLEX™III MALDI-TOF (Bruker Daltonics, Inc.) was used for MS/MS analysis. Tryptic digests were desalted and concentrated with a C4 zip-tip (see protocol, Michrom Biosciences) and applied to the MALDI plate with an α-cyano-4-hydroxycinnammic acid matrix. Laser power was varied to yield maximum resolution in positive reflectron mode. MALDI-MS contains soft ionization properties previously shown to work well with sensitive glycosylation modifications as in this study [9], [17].

## Results

### Identification of EGF-like modules containing an O-β-GlcNAc consensus sequence

**Figure 2:**
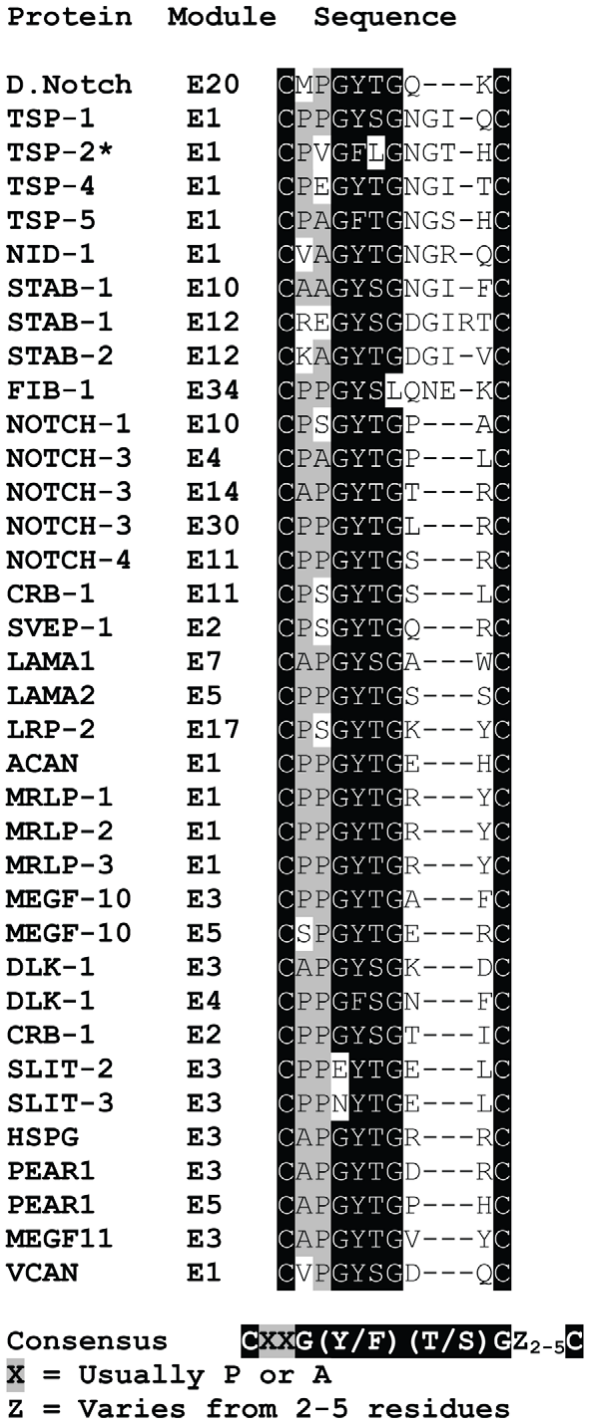
Potential sites of O-β-GlcNAc modification between the fifth and sixth cysteines of EGF-like modules (E) of human proteins. The sequence between the fifth and sixth cysteines of the 20^th^ EGF-like module of *Drosophila* Notch was used as bait in a BLAST search of the human UniProtKB database. The number of the module among the EGF-like modules in a given protein is based on UniProtKB's annotation of the human protein sequences. The E1 module of TSP-2 is also given and discussed in the text.

In the course of examining the extent of modification by glucose (Glc) and xylose (Xyl) on recombinant EGF-like modules of TSP-1 produced in insect High-Five cells [12], we encountered evidence for an adduct of +203-Da, compatible with O-β-GlcNAc. Modification by O-β-GlcNAc of intracellular proteins mediated by cytoplasmic and nuclear O-GlcNAc transferases is well-described [18], [19]. Evidence for lymphocyte cell surface O-β-GlcNAc modification [20] and the recent description of O-β-GlcNAc in the extracellular 20^th^ EGF-like module (E20) of *Drosophila* Notch [9] indicate that extracellular proteins, including those with EGF-like modules, are subject to the modification. When we compared the sequence of *Drosophila* Notch that is modified to the sequence of TSP-1, we found similarities in the loop between the fifth and sixth cysteines of the first EGF-like module, *i.e.*, a serine in TSP-1 and a threonine in Notch are embedded in the sequences PYGSGN and PYGTGQ, respectively (Figure 2). A BLAST search against the human UniProtKB database with the sequence between the fifth and sixth cysteines of the 20^th^ EGF-like module of *Drosophila* Notch as bait revealed that a number of human extracellular matrix and transmembrane proteins in addition to TSP-1 contained the sequence CXXG(Y/F)(T/S)GZ_2–5_C (X typically a Pro or Ala, Z varying from 2–5 residues) between the fifth and sixth cysteines of EGF-like modules (Figure 2). This list includes the first EGF-like modules of TSP-4 and TSP-5, both group B TSPs. The other group A TSP, TSP-2, however, has leucine instead of serine as in TSP-1 (Figure 2).

### Immuno-blotting for the O-β-GlcNAc modification of TSP-1

**Figure 3:**
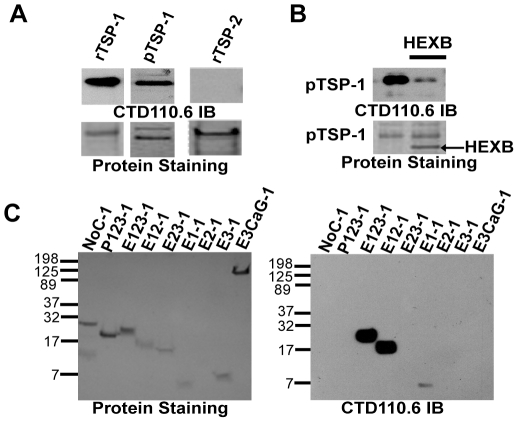
CTD110.6 anti-O-GlcNAc recognition of TSP family of proteins. (A) Anti-O-GlcNAc immuno-blot (IB) and protein staining of recombinant TSP-1 (rTSP-1), platelet TSP-1 (pTSP-1), and recombinant TSP-2 (rTSP-2). Both TSP-1 proteins were positive for CTD110.6 recognition, whereas TSP-2 was negative. Note: the doublet seen with pTSP-1 is a result of the purification from thrombin-stimulated platelets as described previously [26]. (B) Loss of anti-O-GlcNAc immuno-recognition of pTSP-1 after treatment with recombinant β-N-acetyl-hexosaminidase fusion protein (HEXB, MW = ∼100-kDa). (C) Protein staining and anti-O-GlcNAc immuno-blotting of TSP-1 constructs at equal molar concentrations (nomenclature as described in the introduction and Figure 1) to narrow down which TSP-1 modules contain O-β-GlcNAc.

To obtain additional evidence for O-β-GlcNAc modification of TSP-1, monoclonal antibody CTD110.6, which recognizes protein-O-β-GlcNAc conjugates [21], was used to immuno-blot recombinant TSP-1, TSP-1 purified from human platelets, and recombinant TSP-2. TSP-1 purified from human platelets and recombinant TSP-1 reacted with the antibody (Figure 3A). In contrast, recombinant TSP-2, which lacks a modifiable serine or threonine in the hypothesized recognition sequence (Figure 2), did not react (Figure 3A). Immuno-reactivity of recombinant or platelet TSP-1 towards CTD110.6 was diminished after treatment with β-N-acetyl-hexosaminidase (Figure 3B). Inasmuch as CTD110.6 recognizes O-β-GlcNAc and antibody reactivity is sensitive to β-N-acetylhexosaminidase removal of O-HexNAc, i.e., O-β-GlcNAc and O-β-N-acetylgalactosamine adducts, these results indicate that TSP-1, but not TSP-2, is subject to O-β-N-acetylglucosaminylation. To localize the specific module(s) subject to modification in TSP-1, recombinant NoC, P123, E123, E12, E23, E1, E2, E3, and E3CaG constructs (constructs denoted by the nomenclature noted in the introduction and Figure 1) were immuno-blotted with CTD110.6. Only constructs containing the E1 module, which contains the consensus sequence identified between the fifth and sixth cysteines identified in the BLAST search, reacted (Figure 3C).

### Characterization of modification of the TSP-1 E1 module

**Figure 4:**
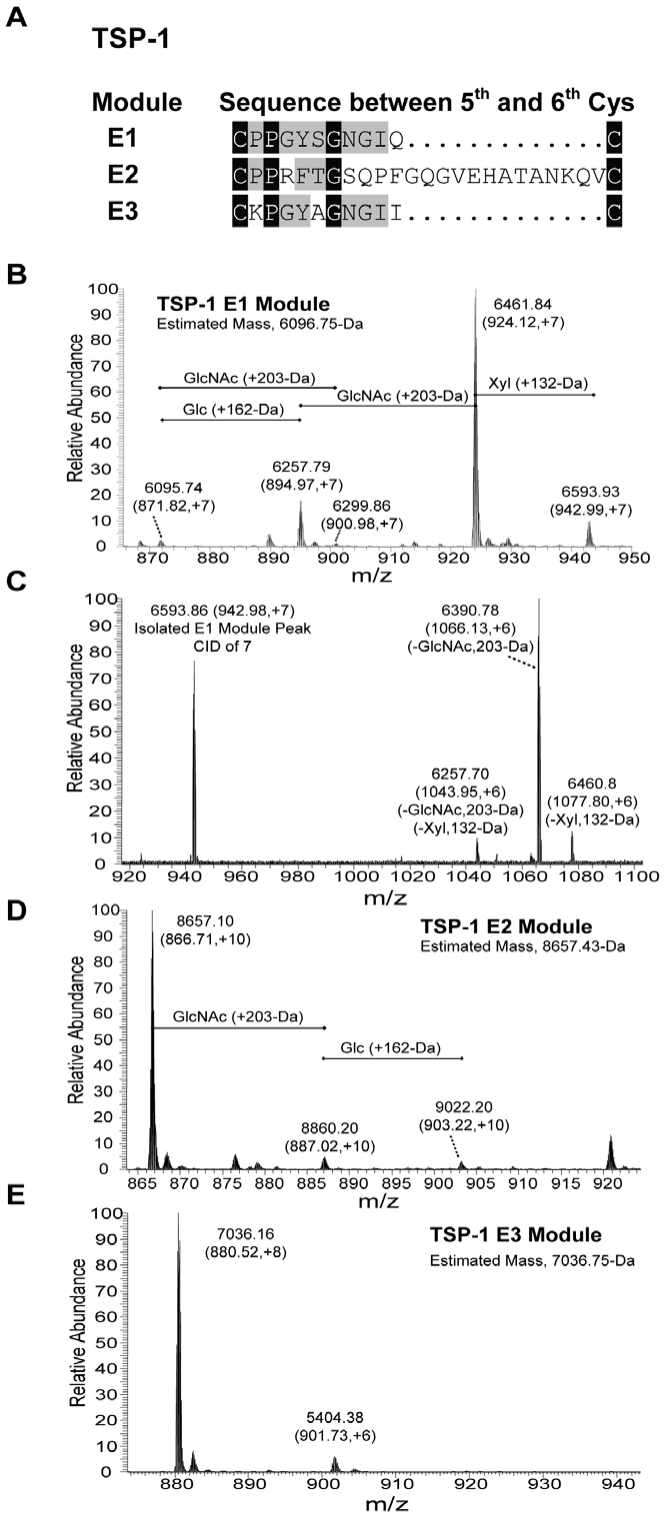
Top-down (LTQ-FT ICR) MS modification analysis of various TSP-1 EGF-like (E) modules. (A) Sequences of the three TSP-1 EGF-like modules are compared. Only E1 contains the O-GlcNAc modification consensus sequence. (B) LTQ-FT ICR MS performed on the TSP-1 E1 module demonstrating modifications in relation to the unmodified precursor size (6096.74-Da). Known Glc (+162-Da) and Xyl (+132-Da) modifications were detected, along with the HexNAc (+203-Da) modification (summed in Table 1). (C) Low energy (q = 7) collision-induced dissociation was then performed on the 6593.93-Da E1 module peak containing Glc, Xyl, and HexNAc from (B) to see if modifications were removed separately. Top-down MS analysis was performed on (D) E2 and (E) E3 modules for comparison.

To corroborate the immuno-blotting results that mapped the potential O-β-GlcNAc modification to E1, we utilized top-down (LTQ-FT ICR) MS modification analysis of the E123, E12, E23, E1, E2, and E3 modular constructs. E2 contains extra residues in the loop between the fifth and sixth cysteines, and E3 lacks a modifiable serine or threonine residue in the loop (Figure 4A). E123, E12, and E1 contained a +203-Da adduct in addition to the known Glc (+162 Da) and Xyl (+132 Da) modification of E1 (Figure 4B and data not shown). Species with the +203-Da adduct accounted for ∼85% of E1 (Figure 4B and Table 1). The 942.99-Da (z = +7) peak, selected because it contains Glc, Xyl, and HexNAc, was isolated during LTQ-FT ICR MS of the E1 module, and collision-induced dissociation at low energy was performed to release the carbohydrates while maintaining integrity of the modular protein. Inspection of the z = +6 ions indicated that Glc and Xyl were removed separately from HexNAc (Figure 4C), indicating that the Glc-Xyl and HexNAc modifications occur at separate sites. LTQ-FT ICR MS analysis of E2 revealed less than 5% was modified with HexNAc, Glc, or Xyl (Figure 4D), and E3 exhibited no detectable modification (Figure 4E).

**Table 1:** LTQ-FT ICR MS quantification of TSP-1 E1 module modifications (Figure 4B).

Glc (+162-Da)	Xyl (+132-Da)	HexNAc (+203-Da)	m/z (+7 ion series)	% of Total
**−**	**−**	**−**	**6095.74**	**1.6**
**+**	**−**	**−**	**6257.79**	**11.6**
**+**	**−**	**+**	**6461.84**	**77.5**
**+**	**+**	**+**	**6593.93**	**8.5**
**−**	**−**	**+**	**6299.86**	**0.8**

Glc indicates glucose; Xyl indicates xylose; HexNAc indicates glucosamine modification.

### MALDI-TOF/TOF MS sequencing of the TSP-1 E1 module

**Figure 5:**
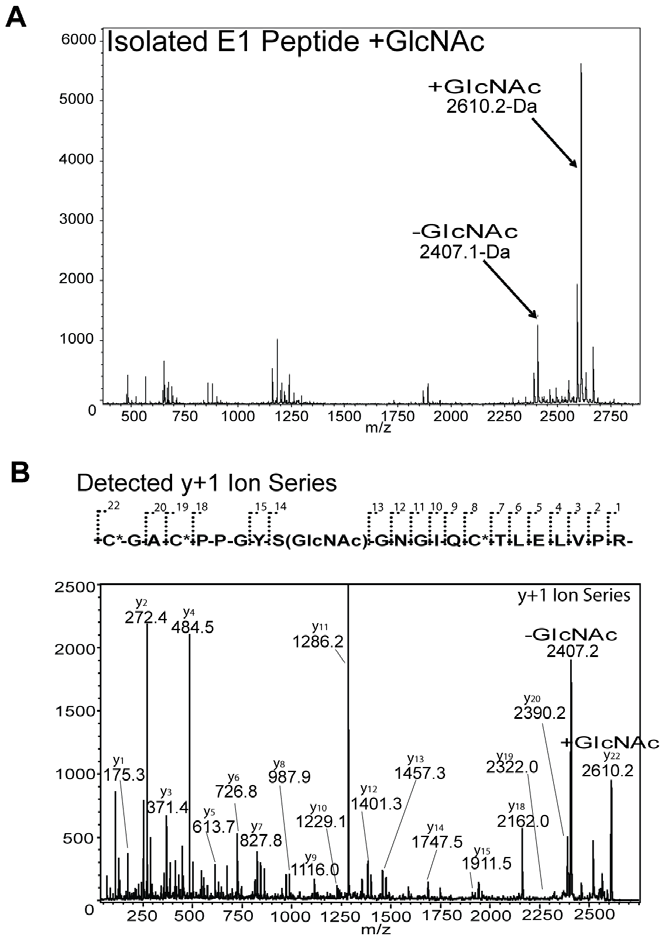
MALDI-TOF/TOF sequencing of O-β-GlcNAc-modified tryptic peptide of the TSP-1 E1 module. The MALDI-TOF spectrum is indicated in (A) with both the −GlcNAc (2407.1-Da) and +GlcNAc (2610.2-Da) E1 module tryptic peptide peaks indicated. The MALDI-TOF/TOF y+1 ion series detected and spectrum are indicated in (B). Note the modification of Ser580 by O-β-GlcNAc from the difference between y_13_ and y_14_ ions. Also, note loss of a +203-Da adduct from a portion of the 2610.2-Da isolated peak and reversion to the unmodified peak as indicated.

To learn if the hypothesized recognition sequence is indeed the site of modification within the E1 module, MALDI-TOF/TOF MS analysis was performed on tryptic digests of reduced and alkylated recombinant E1 module. The CGACPPGYSGNGIQCTLELVPR tryptic peptide, which contains residues 572–587 (underlined) plus part of tail introduced by the expression strategy, was detected as +203-Da modified (2610.2-Da), along with a lesser amount of unmodified peptide (2407.1-Da) (Figure 5A). Isolation of the 2610.2-Da peak, followed by TOF/TOF sequence analysis to identify the site of modification localized the modification to Ser580 confirming O-β-GlcNAc modification (Figure 5B). During this process, some of the isolated 2610.2-Da peptide peak lost the modification, yielding one sequence with Ser580 modified and a second sequence lacking the adduct (Figure 5B).

## Discussion

The O-β-GlcNAc post-translational modification is best known as occurring dynamically on nucleocytoplasmic proteins engaged in a broad range of biological functions [18], [22]. Previous literature, however, indicates that it is an extracellular modification as well [20]. The recent demonstration of O-β-GlcNAc on an EGF-like module of *Drosophila* Notch [9] provided additional evidence that the modification does indeed occur on extracellular proteins. The present study identifies a site of O-β-GlcNAc modification in the 1^st^ EGF-like module of the secreted extracellular matrix protein TSP-1, which shares features with the site of O-β-N-acetylglucosaminylation in Notch. A combination of O-β-GlcNAc specific immuno-blotting, top-down mass spectrometry, and MALDI-TOF/TOF amino acid sequencing demonstrated that Ser580 between the fifth and sixth cysteines of the TSP-1 E1 module contains an O-β-GlcNAc modification. Intracellular GlcNAc modifications occur through cytoplasmic and nuclear O-GlcNAc transferases [18]. The identity of O-GlcNAc transferase(s) that catalyze the modification in secretory organelles is not known. However, previous literature describes membrane protein O-β-GlcNAc modification in the Golgi apparatus [23] as well as at the cell surface [20], [24].

E1 is the only EGF-like module of TSP-1 to have a sequence similar to the sequence for O-β-GlcNAc modification in the Notch receptor. E2 contains serine and threonine residues but there is an extra long 23 residue loop between the fifth and sixth cysteines [8]. We found evidence for minor modification of E2 by O-β-GlcNAc in top-down MS analysis but not by immuno-blotting. The sequence between the fifth and sixth cysteines within the E3 module contains no serine or threonine residue, and no evidence of modification was found by top-down MS analysis. The observations suggest that the short loop between the fifth and sixth cysteines is the preferred site of O-β-N-acetylglucosaminylation.

E1 of TSP-2, which is homologous to E1 of TSP-1, contains a leucine, Leu582, at the position of Ser580 of TSP-1 (Figure 2). Crystallography of a large portion of TSP-2 revealed that Leu582 is in the first strand of a short two-stranded β-sheet that leads into the Ca^2+^-binding site at the interface between E1 and E2 [8]. A BLAST search found numerous other extracellular proteins that contain the apparent consensus sequence for O-β-N-acetylglucosaminylation between the fifth and sixth cysteines of EGF-like modules (Figure 2). Some of the EGF-like modules are predicted to bind calcium; some, like the E1 modules of TSPs [1], are predicted not to bind calcium; and some, like the EGF-like modules of laminins and PEAR-1, are atypical. Thus, there is no obvious common feature beyond the apparent consensus sequence that distinguishes modules containing the sequence or the context in which these modules occur. It should be noted the PG(Y/F)(T/S)G consensus sequence in Figure 2 differs from the (P/V)(P/A)(V/T)(T/S)(T/S) sequence that characterizes sites of O-β-N-acetylglucosaminylation of nucleocytoplasmic proteins [22].

E1 plays a pivotal role in the stalk of TSP-1 by determining the structures of the immediately N-terminal P3 module and immediately C-terminal E2 module [11], [12]. Within a stretch of 83 residues from Trp498 in P3 to Ser580 in E1, all four of the unusual glycosylations found in TSP-1 are present. O-fucosylation of properdin-like modules [6], [7] and O-glucosylation of EGF-like modules [25] are important for maturation and structure of proteins in secretory organelles. We speculate, therefore, that O-β-N-acetylglucosaminylation may be yet another quality control mechanism to insure correct processing and targeted to conformationally labile stretches of modules, such as the P3E12 array of TSP-1, of extracellular matrix or cell surface proteins. In this context, it is interesting that TSP-2, although lacking O-β-N-acetylglucosaminylation because of the presence of Leu582 at the position of Ser580 in TSP-1, is N-glycosylated at Asn584, in the two-stranded β-sheet [8] and that the homologous sequence in TSP-5 contains vicinal sites for potential O-β-N-acetylglucosaminylation and N-glycosylation (Figure 2).
